# Yang-Monti’s Catheterizable Stoma in Children

**DOI:** 10.5812/numonthly.9443

**Published:** 2013-06-25

**Authors:** Rajendra Bapusaheb Nerli, Shivagouda Malgouda Patil, Murigendra Basayya Hiremath, Mallikarjun Reddy

**Affiliations:** 1 Department of Urology, KLES Kidney Foundation, JN Medical College, KLE University, Belgaum, India

**Keywords:** Urinary Bladder, Urinary Diversion, Surgical Stomas, Ileum, Catheterization

## Abstract

**Background:**

In 1981, Mitrofanoff described a procedure to create a continent urinary stoma for clean intermittent catheterization. Since then several procedures have been described including Yang-Monti ileovesicostomy for effective catheterization.

**Objectives:**

We report on our experience from the use of Monti’s procedure in children at our center.

**Patients and Methods:**

Children < 18 years of age undergoing urinary diversion/reconstruction with Yang-Monti’s procedure for congenital conditions or neuropathic bladder formed the study group. All these children, post-operatively were taught clean intermittent catheterization (CIC) and put on a regime using a 14/16 Fr catheter every 3 hours. The children were followed regularly at 3, 6, 12, 18 and 24months post-operatively, with special attention paid to any problems with catheterization and incontinence.

**Results:**

During the period from Jan 2000 to Dec 2011, at our center, 19 children less than eighteen years of age underwent urinary diversion with Yang-Monti’s catheterizable stoma. The indications for urinary diversion was neuropathic bladder in eight, exstrophy bladder in seven , valve bladder syndrome in three and persistent urethral stricture in one. None of the children found CIC difficult during the post-operative period and there was no hindrance to the passage of the catheter.

**Conclusions:**

Although the appendix remains the tissue of choice for creation of catherterizable stoma, the Yang-Monti ileovesicostomy is effective, convenient conduit for children. Long-term complications are minimal and children find this comfortable to do CIC.

## 1. Background

Mitrofanoff (1) made use of the appendix to create a flap valve and promote continence. He believed that any tubular structure could be implanted effectively into a low pressure reservoir to bring about continence. The foundation for the success of this Mitrofanoff principle is based on the creation of a submucosal tunnel for a supple, small diameter conduit. As the reservoir fills, the rise in intravesical pressure is transmitted through the epithelium and to the implanted conduit, coapting its lumen. The appendix is a natural tubular structure which can be removed without significant morbidity. The small diameter of the appendix facilitates the creation of a short functional tunnel with the bladder wall. Kaefer and Retik (2) have shown that continence could be achieved even with a 2 cm appendiceal tunnel. The appendix has been used as an efferent limb with good results, be it with a continent urinary reservoir or with a native bladder (3, 4). The appendix has been particularly useful in children because it is relatively longer and the abdominal wall is generally thinner.

When the appendix is not available for use, then other tubular structures can provide a similar mechanism for catheterization and continence. Mitrofanoff described the use of ureter for similar a purpose (1). Woodhouse and MacNeily (5) used the fallopian tube, which could accommodate catheterization. Bihrle and associates (6) fashioned a gastric tube mobilized on the gastroepiploic artery for implantation in continent reservoirs. Monti and colleagues (7) described a novel modification of the tapered intestinal segment that could be reimplanted according to the Mitrofanoff principle. Yang similarly reported on this procedure (8). This Yang-Monti tube is an excellent example of efficient use of bowel to create a catheterizable stoma (9).

## 2. Objectives

We report on our experience from the use of Monti’s procedure in children at our center.

## 3. Patients and Methods

Children < 18 years of age and undergoing urinary diversion/reconstruction for congenital conditions or neuropathic bladder formed the study group. These children were prospectively assessed and evaluated with biochemical studies of blood and urine. Imaging studies included ultrasonography, x-rays and computerized tomography. Urodynamic studies and Radionuclear studies were done whenever necessary. The need for urinary diversion with catheterizable stoma was planned whenever necessary, after discussion with the children and their parents. Yang-Monti’s tube was planned whenever appendix was short or removed previously.

### 3.1. Surgical Technique

**Figure 1.**
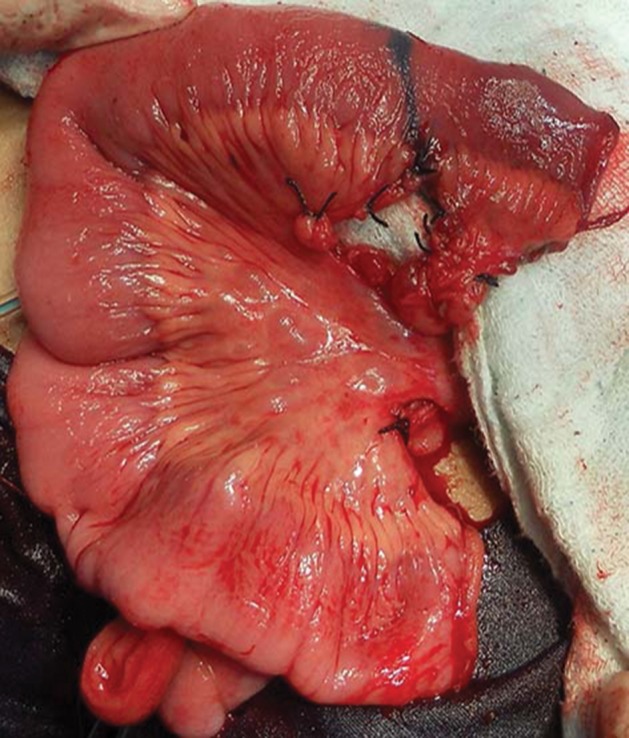
Isolation of 2.5 cm Ileal Segment

**Figure 2.**
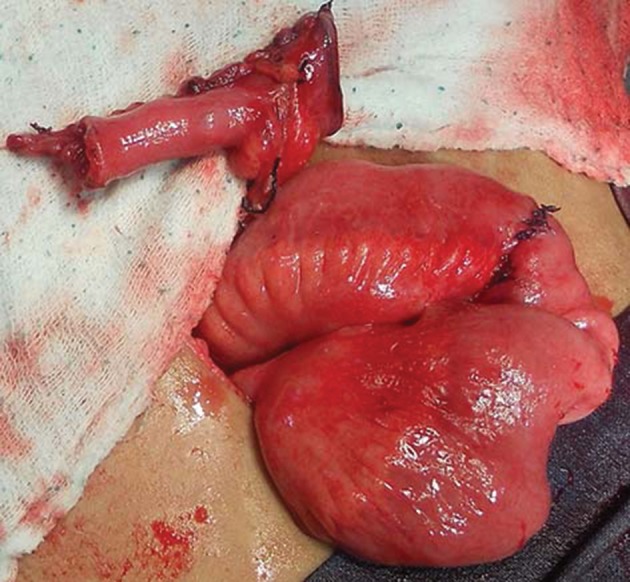
Creation of Yang-Monti’s Tube

**Figure 3.**
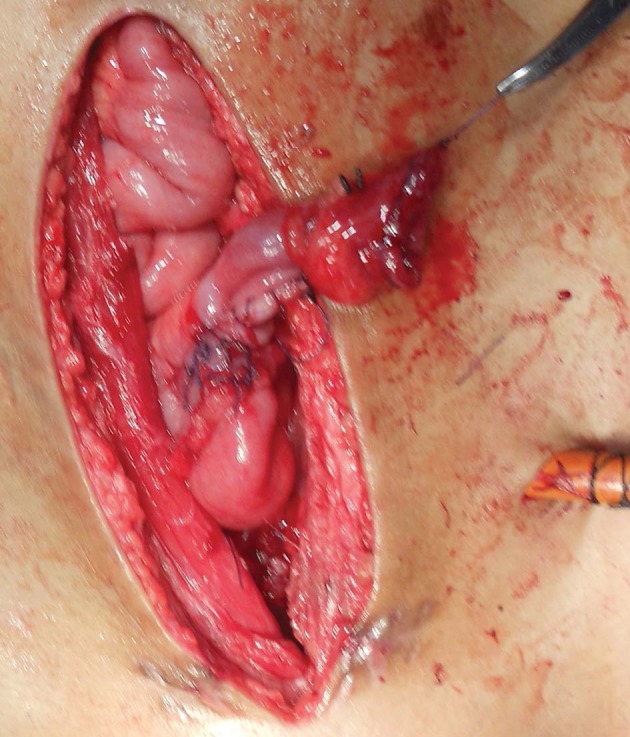
Anastomosis of Yang-Monti’s Tube to the Postero Lateral Aspect of the Augmented Bladder

A 2 to 2.5 cm segment of ileum with a suitable blood supply was isolated (Figure 1), opened along its anti-mesenteric border and then closed transversely surrounding a 14-16 Fr urethral catheter using running Vicryl sutures. The diameter of the small intestine determined the length of the newly built tube. Preferably the terminal 15 cm of the ileum was not used to isolate the intestinal segment. Whenever a longer catheterizable conduit was necessary, a 3.5 to 4 cm section of ileum was isolated on its mesentery. The bowel was divided into two equal segments for approximately 80% of its circumference, leaving the bowel intact over the mesentery. Each ring of bowel was incised adjacent to the mesentery, but on the opposite sides, so as to allow the bowel rings unfold in opposite directions. The unfolded bowel was straightened and tubularized along the long axis (Figure 2). This ileal tube was then anastomosed to the superior part of the postero-lateral junction of the bladder (Figure 3).

All children were discharged 5-6 days post-operatively, as soon as they could tolerate solid food. The diversion catheter was removed 2 weeks following surgery. All these children were taught clean intermittent catheterization (CIC) and put on a regime using a 14/16 Fr catheter every 3 hours. Presence of urinary leakage during this interval was considered as incontinence. The children were followed regularly at 3, 6, 12, 18 and 24 months post-operatively, with special attention paid to any problems with catheterization and incontinence.

## 4. Results

During the period between Jan 2000 to Dec 2011, at our center, 19 children less than eighteen years of age underwent urinary diversion with Yang-Monti’s catheterizable stoma. The mean age of the children was 12.1 years. Twelve of these children were boys and the remaining seven girls .The indications for urinary diversion (Table 1) was neuropathic bladder in eight, exstrophy bladder in seven, valve bladder syndrome in three and persistent urethral stricture in one. 

Of the 8 children with neuropathic bladders, six of them had myelodysplasia and two developed neurogenic bladders following trauma. Of these eight children, four needed augmentation as they had a poorly complaint bladder, with bilateral hydronephroureterosis and were presented with severe incontinence. In all these eight children it was easily possible to create a Yang-Monti’s tube up to the umbilicus for self catheterization.

Seven children had an exstrophy bladder. Four of these had undergone closure of the exstrophy in early childhood, whereas the remaining three had not undergone any repair. They were all presented with total incontinence. All the four children who had undergone repair, had multiple vesical calculi, a small capacity thickened bladder and hydronephroureterosis. In 3/7 children the small fibrotic bladder was excised and a continent pouch with Yang-Monti’s catheterizablestoma was created. In the remaining four, the bladder patch was used as a portion of the continent pouch with catheterizable stoma.

Three children with a past history of posterior urethral valves also underwent augmentation cystoplasty with catheterizable stoma. All these three children were incontinent before surgery, and had raised renal parameters. These children were put on anticholinergics and a strict regime of CIC after urodynamic studies and in the pre-operative period. The serum creatinine levels were stabilized to < 0.6 in all three patients.

One child who had severe urethral distraction injury following a road traffic accident, had previously undergone three repairs at various centers. After evaluation and discussion, surgery was performed on this child for the creation of Yang-Monti’s catheterizable stoma for CIC. There was no dehiscence, necrosis or perforation of the tube in any of the children. All these children were able to successfully perform CIC at the required time.

During the follow-up period ranging from 13 to 66 months (mean 43 months) it was noted that all these children performed CIC effectively. Two children developed stomal narrowing. One could be easily managed with dilatation, whereas the other child needed refashioning of the stoma, two years after the initial surgery. None of the children found CIC difficult and there was no hindrance to passage of the catheter. One child continues to have occasional nocturnal incontinence.

**Table 1.** Indications for Yang Monti’s Procedure

No	Indication	No. of Children
**1**	Neuropathic bladder	8
**2**	Exstrophy bladder	7
**3**	Valve bladder syndrome	3
**4**	Persistent urethral stricture	1

## 5. Discussion

Mitrofanoff’s contribution for the development of extra anatomical continent stoma for intermittent bladder catheterization stands as a great step forward in the surgical treatment of congenitally malformed and neuropathic bladders (1). The Mitrofanoff principle has evolved to include appendicovesicostomy, catheterizableureterostomy, continent vesicostomy and reconfigured ileovesicostomy (5, 7, 10). Several authors believe that although the appendix remains the tissue of choice for the construction of a continent catheterizableconduit, the Monti’s procedure has substantial advantages over other efferent catheterizable tubes. These include the need for a very short segment of the bowel (2.5 cm), adequate lumen size (16 to 18 F), length, reliable blood supply, and the versatility to combine with a simultaneous bowel patch in the same pedicle for bladder augmentation (11).

**Figure 4.**
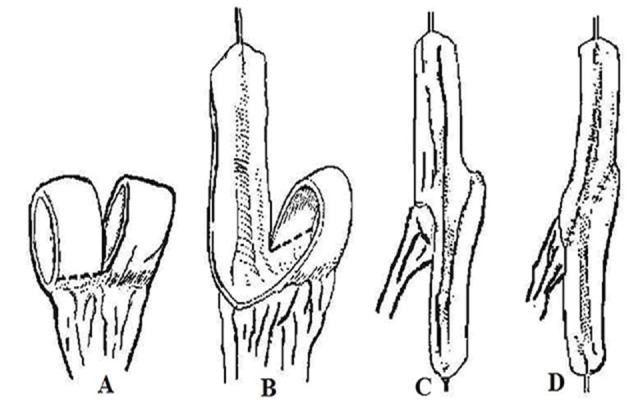
Diagrammatic Representation of Construction of Ileal Tube A. 3.5 cm ileal segment is isolated on its mesentery. B. segment is divided in half for an 80% of circumference, leaving strip of bowel intact over mesentery, resulting 2 strips of ileum are incised and opened on opposite sides, leaving strip intact over the mesentery. C. incised rings are unfolded in opposite directions, unfolded bowel is straightened and reconfigured by sewing corners along the mesentery to main strip. D. long strip of bowel is rolled into a tube over a catheter; the resulting tube is 12 to 14 cm long and accepts a 10F catheter. Mesentery is in the center. Either end may be extensively spatulated or shortened as needed.

One of the potential disadvantages of the Yang-Monti tube is that it remains relatively short and may not reach the skin without tension in obese patients .The length of these ileovesicostomies is limited by the circumference of the bowel segment used, which is inadequate in some cases. Similarly in children with spinal abnormalities or simply a long distance from the native bladder to the umbilicus the distance exceeds the length of available tube. In such situations, the surgeon must place the stoma in a less than desirable location, reimplant the tube into the bowel segment or use 2 ileal segments in sequence. Casale (12) described a procedure to double the length of the Yang-Monti ileovesicostomy using a single section of the bowel. A 3.5 cm section of ileum is isolated on its mesentry, the bowel is divided into 2 segments for 80% of its circumference, leaving the bowel intact over the mesentry. Each ring of the bowel is then divided adjacent to the mesentry but on opposite sides, allowing the bowel to be unfolded and reconfigured in a single long strip that may then be tubularized. The blood supply to the tube is excellent and it is in the center of the reconfigured ileum. The ends may be trimmed or widely spatulated as necessary (Figure 4).

Catheterization of this Yang-Monti tube should be easier as the circular mucosal folds are redirected longitudinally in the direction of catheter passage. However Narayanaswamy and colleagues (13) noted difficulty with catheterizations through Yang-Montiileovesicostomy in 7 (28%) of their patients due to pouch like dilatation. Two of these patients needed resection of these pouches. Kaefer et al. (14) believe that stomas constructed from the ileum may have a lower rate of stomal stenosis than those made from the appendix. In our series too, the children were able to perform CIC comfortably and effectively.

Although the appendix remains the tissue of choice for the creation of catherterizable stoma, the Yang-Monti ilevesicostomy is an effective and convenient conduit in children. Long term complications are minimal and the children find this comfortable to do CIC.
